# Comparative secretome analysis of *Striga* and *Cuscuta* species identifies candidate virulence factors for two evolutionarily independent parasitic plant lineages

**DOI:** 10.1186/s12870-024-04935-7

**Published:** 2024-04-06

**Authors:** James M. Bradley, Roger K. Butlin, Julie D. Scholes

**Affiliations:** 1 School of Biosciences, University of Sheffield, Western Bank, Sheffield, S10 2TN UK; 2 Present address: Department of Cell and Systems Biology, University of Toronto, Toronto, ON M5S 3B2 Canada; 3 Department of Marine Sciences, University of Gothenburg, 405 30 Gothenburg, Sweden

**Keywords:** Host-parasite interaction, Parasitic plants, *Striga*, *Cuscuta*, Virulence factor, Secretomes, Transcriptomics

## Abstract

**Background:**

Many parasitic plants of the genera *Striga* and *Cuscuta* inflict huge agricultural damage worldwide. To form and maintain a connection with a host plant, parasitic plants deploy virulence factors (VFs) that interact with host biology. They possess a secretome that represents the complement of proteins secreted from cells and like other plant parasites such as fungi, bacteria or nematodes, some secreted proteins represent VFs crucial to successful host colonisation. Understanding the genome-wide complement of putative secreted proteins from parasitic plants, and their expression during host invasion, will advance understanding of virulence mechanisms used by parasitic plants to suppress/evade host immune responses and to establish and maintain a parasite-host interaction.

**Results:**

We conducted a comparative analysis of the secretomes of root (*Striga* spp.) and shoot (*Cuscuta* spp.) parasitic plants, to enable prediction of candidate VFs. Using orthogroup clustering and protein domain analyses we identified gene families/functional annotations common to both *Striga* and *Cuscuta* species that were not present in their closest non-parasitic relatives (e.g. strictosidine synthase like enzymes), or specific to either the *Striga* or *Cuscuta* secretomes. For example, *Striga* secretomes were strongly associated with ‘PAR1’ protein domains. These were rare in the *Cuscuta* secretomes but an abundance of ‘GMC oxidoreductase’ domains were found, that were not present in the *Striga* secretomes. We then conducted transcriptional profiling of genes encoding putatively secreted proteins for the most agriculturally damaging root parasitic weed of cereals, *S. hermonthica*. A significant portion of the *Striga*-specific secretome set was differentially expressed during parasitism, which we probed further to identify genes following a ‘wave-like’ expression pattern peaking in the early penetration stage of infection. We identified 39 genes encoding putative VFs with functions such as cell wall modification, immune suppression, protease, kinase, or peroxidase activities, that are excellent candidates for future functional studies.

**Conclusions:**

Our study represents a comprehensive secretome analysis among parasitic plants and revealed both similarities and differences in candidate VFs between *Striga* and *Cuscuta* species. This knowledge is crucial for the development of new management strategies and delaying the evolution of virulence in parasitic weeds.

**Supplementary Information:**

The online version contains supplementary material available at 10.1186/s12870-024-04935-7.

## Background

Parasitic plants have repeatedly evolved from autotrophic plants, and are defined by their ability to infect, and gain sustenance from, other plants [1]. Parasitic plants display various degrees of reliance on their host plant, ranging from complete holoparasitism (all nutrition derived from the host) to hemiparasitism (some nutrition derived from host plants and some through their own, limited photosynthesis) [1, 2]. Parasitic plants can be further classified by whether they infect the root or shoot of their host plant and may form vascular connections with the host xylem and/or phloem [3, 4]. Hemiparasitic *Striga* spp. (Orobanchaceae family) parasitize roots and connect only with the host’s xylem [3, 4], whilst holoparasitic *Cuscuta* spp. (Convolvulaceae family) parasitize host shoots and form direct connections with the host’s phloem and xylem [5]. Both these genera of parasitic plants severely constrain agriculture on the African continent [4, 6, 7].

Despite diversity in evolutionary origins and mode of parasitism, all parasitic plants develop an haustorium, the infection organ through which the parasite attaches to the host, forms direct connections with the vasculature, and withdraws water and nutrients [1, 8]. The haustorium also provides a route for delivery of molecules (e.g. small RNAs, proteins and/or hormones) from parasitic plants into their host plants [8–11]. In other plant-parasite interactions, such as those involving fungi, oomycetes or nematodes, research has identified numerous parasite-derived secreted protein virulence factors (VFs) that the parasite delivers into the host to manipulate its cellular machinery, thus facilitating colonization and parasitism [12–16]. Plants have evolved to detect parasite VFs either directly or indirectly (via their damage inflicted on the host) and to trigger a defence response [15, 17, 18].

As with any plant parasite, parasitic plants must achieve two goals to successfully infect a host plant: (i) overcome primary physical host defences (e.g. the suberised endodermal cell walls); and (ii) avoid triggering, or actively suppress, a host defence response [11]. It is now recognised that parasitic plants achieve these goals in part through the deployment of secreted VFs [11, 19, 20]. Examples include proteins that loosen host cell walls, such as pectin methylesterases or expansins, and those that interact with components of the host immune system to suppress a defence response [8, 9, 11, 19, 21–23]. For instance, Su et al*.* (2019) [19] demonstrated that a particular ‘race’ of *Striga generioides* secretes a leucine-rich repeat (LRR) domain-containing VF, called Suppressor of Host Resistance 4z (SHR4z), into host cells. It then triggers turnover of the E3 ubiquitin ligase, *Vu*POB1, leading to suppression of host plant immunity.

Often, as is the case for SHR4z, VFs have an N-terminal secretion signal to direct the protein out of the parasite’s cell into the host plant [19]. Although not all VFs possess N-terminal secretion signals, nor are all secreted proteins VFs, the ease and high-throughput nature of N-terminal secretion signal detection makes this an attractive starting point to identify and compare the comprehensive secretomes of multiple species as a step towards identifying putative VFs. Secretome predictions have been conducted for numerous plant parasites [24–28], including *S. hermonthica* where it was recently used to complement a population genomics experiment that identified candidate secreted VFs from this parasitic plant [20]. Although studies have revealed VFs from a range of parasitic plants (e.g. [19, 20, 23]), their discovery and mechanism of action lags far behind that of other plant pathogens such as nematodes and filamentous fungi. Moreover, no comprehensive secretome comparisons have yet been conducted between different parasitic plant species or families.

The main aim of this study was to conduct a comparative analysis of the secretomes of root (*Striga* spp.) and shoot (*Cuscuta* spp.) parasitic plants, to enable prediction of candidate VFs. We used recently published genomes of 4 parasitic plant species to predict the secretomes for *Striga* (*S. hermonthica* and *S. asiatica*) and *Cuscuta* (*C. campestris* and *C. australis*) species. We did the same for two closely related non-parasitic plant species (*Mimulus guttatus* and *Ipomea nil*, respectively). We then conducted gene clustering and protein domain analyses to identify protein families / domains associated with the secretomes of either parasitic genera, which we called *Striga*- or *Cuscuta*-specific. This analysis revealed similarities and differences in candidate VFs in the *Striga* and *Cuscuta* species. For example, we found PAR1 protein domains and GMC oxidoreductase domains were specifically associated with either the *Striga* or *Cuscuta* secretomes, respectively.

In order to identify genes from our *Striga*-specific secretome set that were differentially expressed during parasitism of a susceptible host, we first profiled the expression of all genes encoding secreted proteins in *S. hermonthica*. We collected S. *hermonthica* haustoria attachments at 2-, 4- and 7-days post infection (dpi) of a susceptible host rice cultivar, NERICA 7. We specifically focused on the genes encoding putative secreted proteins that showed differential expression during host invasion. Some of these genes are likely to represent VFs, given that the expression of such genes is typically coordinated with host invasion in other parasite-host systems (e.g. [29, 30]).

We found that a highly significant portion of our *Striga*-specific secretome set were differentially expressed during parasitism, including an abundance of cell wall modifying enzymes and leucine rich repeat containing proteins. This strongly corroborates a recent population genomics-based analysis aimed at discovering VFs from this species, and a study that identified an effector from a closely related parasitic plant, *S. gesnerioides* [19, 20]. We further probed this dataset to identify those genes following a ‘wave-like’ pattern of expression that peaked at the early penetration stage of infection, just as the parasite penetrates the cortex of the host root, characteristic of VFs. We identified 39 putative VFs of *S. hermonthica* that are excellent candidates for future functional studies.

## Results

### Overview of the predicted plant secretomes

**Fig. 1:**
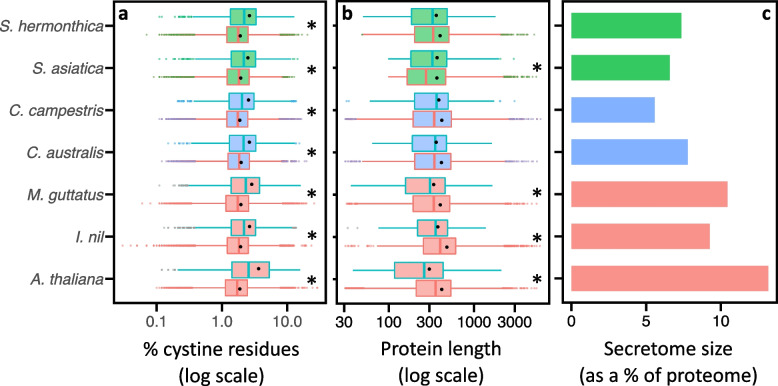
Characteristics of the plant secretomes assessed in this study. **a**-**b** Boxplot distributions of protein cysteine content (**a**) and length (**b**) for secreted proteins (blue boxes) and non-secreted proteins (red boxes). For each distribution, the centre line in each box indicates the median and black dots indicate the mean. Asterisks (*) indicate the medians for the secretome and non-secretome distributions were significantly different (*p* < 0.001, Wilcoxon rank sum test). **c** The secretome sizes as a percentage of the proteome. Red: non-parasitic plants. Green: root parasitic plants. Blue: shoot parasitic plants

We predicted the secretomes of seven plant species. In all cases, the secretomes were enriched for proteins with more cysteine residues compared to non-secreted proteins, which is a common feature for secreted proteins that is thought to confer protein stability in the extracellular environment (Fig. 1a). All four parasitic plant species had smaller secretomes (as a percentage of the proteome) compared with the autotrophic plants (Fig. 1c). Furthermore, all three autotrophic plants had secretomes consisting of proteins that were on average smaller in length than the non-secreted proteins (red boxes in Fig. 1b). In contrast, the distribution of protein length for the parasitic plant secretomes was either no different from the non-secreted proteins or, in the case of *S. asiatica*, protein length for secreted proteins was on average significantly longer than the non-secreted proteins (Fig. 1b). Thus, for the parasitic plant species analysed in this study, there was a trend towards smaller secretomes, which on average consisted of larger proteins (Fig. 1b, c). Intriguingly, *C. campestris* had the smallest secretome size overall, despite a recent whole genome duplication [31] (Fig. 1c).

### Comparative analysis of the *Striga* and *Cuscuta* secretomes

**Fig. 2:**
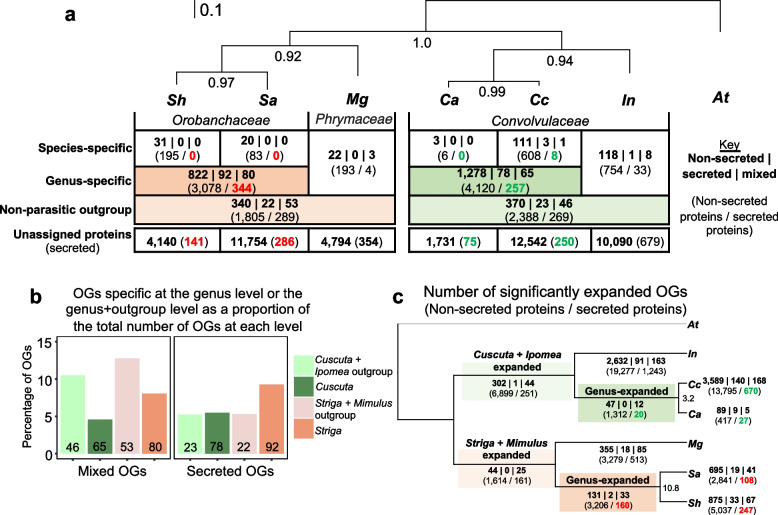
Clustering of secreted and non-secreted plant proteins into orthogroups reveals species-specific and genus-specific gene families for shoot and root parasitic plants. **a** A STAG (Species Tree inference from All Genes) species tree inferred from single-copy orthogroups (OGs). The STAG support values at internal nodes denote the proportion of times that the bipartition was observed for each species tree estimated from each OG. Scale denotes the average number of amino acid substitutions per site. *Sh: Striga hermonthica, Sa*: *Striga asiatica, Mg*: *Mimulus guttatus, Ca*: *Cuscuta australis, Cc*: *Cuscuta campestris,* In: *Ipomoea nil, At*: *Arabidopsis thaliana*. The table shows the number of OGs specific to either the species, or genus, or at the level that includes the non-parasitic outgroup. OG counts are divided into three groups: consisting entirely of non-secreted proteins (non-secreted), entirely of secreted proteins (secreted) or a mix of non-secreted and secreted proteins (mixed). **b** The proportion of secreted or mixed OGs at the genus and non-parasitic outgroup level. Counts are given at the base of each bar. **c** An ultrametric tree for the plant species used in this study showing the numbers of significantly expanded OGs for each branch. The numbers in bold denote the number of expanded OGs for each branch. For a and c, the numbers in parenthesis represent the numbers of non-secreted or secreted proteins found in all the OG at each level. Numbers highlighted red or green represent the putative secreted proteins found in OGs that were either specific to (**a**) or expanded in (**c**) the *Striga* or *Cuscuta* genera, respectively

To gain a broad perspective on how the secretome of *Striga* species (*S. hermonthica* and *S. asiatica*) coincides with *Cuscuta* species (*C. campestris* and *C. australis*), we conducted a comparative orthogroup analysis. In addition, the non-parasitic close relatives of the *Striga* and *Cuscuta* genera were included (*M. guttatus* and *Ipomea nil*, respectively), as was *Arabidopsis*. Initially, the entire proteomes of these seven plant species were clustered into orthologous groups, or orthogroups (OGs). The *M. guttatus* and *I. nil* proteomes provided non-parasitic outgroups to the *Striga* and *Cuscuta* genera, respectively, and allowed shared and lineage-specific protein families to be identified between the *Striga* and *Cuscuta* species / genera (Fig. 2a). Thus, OGs containing only sequences from the *Striga* spp. and none from *M. guttatus*, were deemed *Striga*-specific, and OGs with sequences from only *Cuscuta* spp. and none from *I. nil* were deemed *Cuscuta*-specific (Fig. 2a).

Overall, 18,458 OGs were identified among the seven plant species, 375 of which were species specific, 287 were single-copy OGs (exactly one sequence from each species) and 9,187 had sequence membership from all species (Additional File 1). A phylogeny built using the 287 single-copy OGs revealed longer branch lengths (representing the proportion of amino acid changes per site) for the parasitic plant species, compared with their non-parasitic relative (see tree in Fig. 2a), indicating that these species have experienced more rapid rates of evolution.

Most proteins from each species were assigned to OGs (between 66–90.5% of the proteome), with the remaining being unassigned to any OG (Fig. 2a, Additional File 1). We further defined OGs based on whether they contained only non-secreted proteins (non-secreted OGs), only secreted proteins (secreted OGs) or a mixture of the two (mixed OGs) (Fig. 2a). A small number of the OGs were specific to a single parasitic plant species, but these almost entirely comprised non-secreted OGs and inspection of the functional annotations for these proteins revealed a propensity of transposon-related functional annotations (e.g. PF10551: MULE transposase domain, PF14223: gag polypeptide of LTR copia type, and PF08284: retroviral aspartyl protease) or proteins with no known Pfam domain (Additional File 2). In contrast, OGs that were genus-specific (i.e. OGs with sequences from both *Striga* spp. or both *Cuscuta* spp. but not from any other species), included many secreted and mixed OGs (Fig. 2a, Additional File 2).

We predicted that the transition to parasitism might be associated with an increase in the types and abundances of secreted proteins. If true, we expected secreted and/or mixed OGs would be over-represented among OGs specific to parasitic genera but not at the level that includes the non-parasitic relative. We tested this prediction by comparing the observed numbers of OGs for our *Striga* and *Cuscuta* parasitic genera to the numbers seen at the non-parasitic outgroup level (Fig. 2b, Additional File 3). For instance, of the 994 *Striga*-specific OGs, 9% were secreted OGs and 8% mixed OGs, whilst at the non-parasitic outgroup level there were 415 OGs, comprising 5% secreted and 13% mixed OGs (Fig. 2b). Thus, the parasitic *Striga* genus had a significant enrichment of secreted OGs and a depletion of mixed OGs relative to the non-parasitic outgroup level (Chi-squared statistic = 12.6, *p*-value = 1.84E^−03^; Fig. 2b; Additional File 3). For the parasitic *Cuscuta* genus, there was also a significant depletion of mixed OGs at the genus level (Chi-squared statistic = 20.8, *p*-value = 2.98E^−05^), but no difference in the proportion of secreted OGs, relative to the non-parasitic outgroup level (Fig. 2b). Thus, although there is some evidence for an enrichment of secreted OGs in the *Striga* genus, this was not true for *Cuscuta* or for mixed OGs, contrary to expectation.

In addition to those OGs specific to each parasitic plant genus, we also defined OGs that were not specific to a genus but were significantly expanded along the branches of the tree leading to each parasitic plant (Fig. 2c; Additional File 4). The numbers of significantly expanded OGs were similar for *S. hermonthica* (975) and *S. asiatica* (755) (Fig. 2c). However, the 975 expanded OGs identified in the branch of the phylogeny leading to *S. hermonthica* contained almost double the number of proteins (5,284) than for the branch leading to *S. asiatica* (2,949), which could be explained by whole genome duplication event in the *S. hermonthica* lineage [32]. Similarly, a whole-genome duplication has been suggested to have occurred in the lineage leading to *C. campestris* [31], which could explain the many more significantly expanded OGs in *C. campestris* compared with *C. australis* (Fig. 2c).

Given the independent evolutionary origins of parasitism in the *Striga* and *Cuscuta* genera, we predicted it would be unlikely to find OGs consisting entirely of proteins from *Striga* and *Cuscuta* parasites without representative sequences from the non-parasitic relatives. Unexpectedly, however, we found 85 OGs that contained protein sequences from at least three of the four parasitic plant species (17 of these OGs had sequences from all four parasitic plant species), without any sequence from either of the closely related non-parasitic plants (bold numbers in Fig. S1, Additional File 5). Of these 85 OGs, 11 contained putative secreted proteins, five of which lacked any known Pfam domain and were of unknown function, whilst two had protein domains indicative of cell wall modification (pectinesterase and a lytic transglycolase) (Table 1). Most notably, one of these OGs (OG0011191) annotated with the Pfam domain ‘strictosidine synthase’, consisted of eight protein sequences (including seven putative secreted proteins) and had representative sequences from all four parasitic plant species (Table 1). This prompted us to look at all the OGs containing proteins annotated with the Pfam domain: PF03088–strictosidine synthase. We found seven OGs that contained protein sequences with this domain from at least one parasitic plant (Fig. S2). However, only OG0011191 (Table 1) contained sequences exclusively from the parasitic plants, without any sequences from the autotrophic plants (Table 1; Fig. S2). Thus, the *Striga* and *Cuscuta* protein sequences in OG0011191 are sufficiently different from all other strictosidine synthase like sequences, even from the non-parasitic close relatives, to cluster together into a single protein family. There were also other OGs with protein sequences from the four parasitic plants species and none from either *M. guttatus* or *I. nil* that did not contain putative secreted proteins. Many were annotated with F-box-associated domain (OG0000693 and OG0004909) or a proteosome subunit (OG0009220) (Additional File 5).

**Table 1:** The 11 orthogroups which contained putative secreted protein sequences from at least three of the four parasitic plants and which lacked any sequence from the close-relatives *Mimulus guttatus* or *Ipomea nil*. Numbers represent the number of protein sequences for each parasitic plant (number of which that were predicted to be secreted in brackets). The most frequently found Pfam domain among all the protein sequences for each OG is also shown

Orthogroup	*Sh*	*Sa*	*Cc*	*Ca*	Most frequent Pfam domain in OG
OG0011275	2 (0)	2 (1)	3 (0)	1 (0)	PF 14541–Xylanase inhibitor C-terminal
OG0011191	1 (0)	1 (1)	4 (4)	2 (2)	PF03088–Strictosidine synthase
OG0013739	1 (0)	1 (1)	2 (0)	1 (0)	No Pfam
OG0013776	1 (1)	1 (1)	2 (1)	1 (1)	PF00067–Cytochrome P450
OG0015488	1 (1)	0 (0)	1 (1)	1 (0)	PF00188–Cysteine-rich secretory protein family
OG0000108	0 (0)	2 (0)	48 (1)	8 (0)	No Pfam
OG0012683	2 (2)	1 (1)	1 (1)	0 (0)	No Pfam
OG0011886	1 (0)	4 (1)	1 (0)	0 (0)	No Pfam
OG0016209	1 (1)	1 (0)	1 (0)	0 (0)	No Pfam
OG0014899	1 (0)	1 (1)	2 (0)	0 (0)	PF01095–Pectinesterase
OG0014352	1 (1)	1 (0)	0 (0)	2 (0)	PF01357;PF03330–Pollen allergen: Lytic transglycolase

*Sh: Striga hermonthica*, *Sa: Striga asiatica*, *Cc: Cuscuta campestris*, *Ca: Cuscuta australis*

In addition to OG clustering, we identified significantly enriched Pfam domains in the secretomes, relative to the rest of the proteome. The number of enriched domains ranged from 187–215, of which 111 were found to be enriched in the secretomes of all plant species (excluding *A. thaliana*) (Additional File 6). Subsets of domains were enriched only in one parasitic plant species, or only in the *Striga* or *Cuscuta* genera (Additional File 6). These domains typically had low abundances in their respective secretomes, accounting for < 0.25% of all domains in the secretome. There were two exceptions to this. First, a cytochrome P450 domain (PF00067) was enriched only in the secretomes of the two *Striga* spp. with an abundance of 1.3% and 1.0% in the *S. hermonthica* and *S. asiatica* secretomes, respectively (Additional File 6). Second, a leucine rich repeat domain (PF13516) was enriched only in the secretomes of the two *Cuscuta* spp. with a domain abundance of 0.6% and 0.9% in the *C. campestris* and *C. australis* secretome, respectively (Additional File 6).

**Fig. 3:**
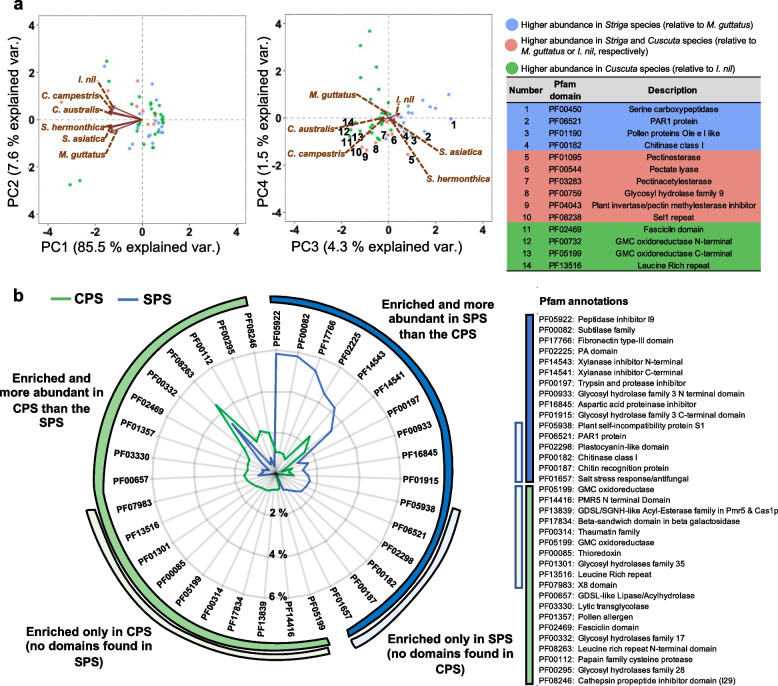
Clustering secretome enriched protein domains revealed distinct and common functionalities associated with root and shoot parasitic plant secretomes. **a** Principal component (PC) biplots for secretome enriched Pfam domains. PC scores for each Pfam domain (data points) and the loadings for each species (arrows) are given for PC 1–4. Table insert provides Pfam descriptions for selected domains labelled in the biplot for PC 3 and 4 (complete data set in Additional File 7). **b** Significantly enriched Pfam domains found within secreted proteins with the biggest difference in abundance between the *Striga* parasite set (SPS) and *Cuscuta* parasite set (CPS) of proteins (as defined in the methods section). Only the 35 most different are shown (complete data set in Additional File 8)

To identify domains that were not necessarily enriched specifically in one parasitic plant secretome, but which were still associated with the secretomes of a parasitic plant, a principal component analysis (PCA) was conducted using the Pfam abundances as input (Fig. 3a, Additional File 7). The second principal component separated the Orobanchaceae and Convolvulaceae families in PCA space (Fig. 3a). The third and fourth principal components explained a small portion of the variation (4.3% and 1.5%, respectively) but separated the parasitic plants from their non-parasitic relatives (Fig. 3a). Those domains that were most strongly associated with *Striga* spp. (but not *M. guttatus*) included the serine carboxypeptidase, PAR1 and pollen protein Ole domains, whilst two domains associated with GMC oxidoreductase were more strongly associated with *Cuscuta* spp. secretomes (but not *I. nil*) (Fig. 3a). Finally, a suite of protein domains involved in pectin modification (pectinesterase, pectate lyase, pectinacetylesterase and plant invertase/pectin methylesterase inhibitor) were identified for their high abundance in the secretomes of both the *Cuscuta* and *Striga* spp., relative to the non-parasitic relatives (Fig. 3a, Additional File 7).

Given the close evolutionary relationship between parasitic plants and their host plants (relative to other plant parasites (e.g., fungi and nematode parasites)), it is challenging to identify which proteins within parasitic plant secretomes represent possible VFs and which participate in other aspects of the parasitic plant’s biology. To address this challenge, we used our orthogroup analysis to generate subsets of *Striga* or *Cuscuta* proteins that were more distinct from the closely related non-parasites. Proteins were assigned into a *Striga* Parasite Set (SPS) or a *Cuscuta* Parasite Set (CPS) if the protein either: (i) did not have a corresponding ortholog in another plant species from this study; (ii) was found in an OG that was expanded in a parasitic plant lineage relative to their non-parasitic plant relative; or (iii) was unassigned to an orthogroup. This led to the identification of 1,125 proteins within the SPS (sum of the red numbers in Fig. 2a and c, after removal of duplicates) and 1,220 proteins within CPS (sum of the green numbers in Fig. 2a and c, after removal of duplicates; Additional File 8).

We then determined enriched Pfam domains for these two protein sets and plotted these according to those which showed the largest difference in abundance (Fig. 3b). For example, the peptidase inhibitor I9, subtilase family, fibronectin type-III and PA domains were all very abundant in the SPS compared to their abundances in the CPS (Fig. 3b). Intriguingly, these four domains often appeared together in the same protein, forming a multi-domain protein. Whereas, a different peptide inhibitor domain, the cathepsin propeptide inhibitor domain (I29), was more abundant in the CPS compared with the SPS (Fig. 3b). There were also several domains enriched only in the SPS or the CPS. For example, the PAR1 domain was exclusively enriched in the SPS, whilst the GMC oxidoreductase domain was exclusive to the CPS (Fig. 3b).

### Transcriptome profiling of the *S. hermonthica* secretome during parasitism of a susceptible rice host

**Fig. 4:**
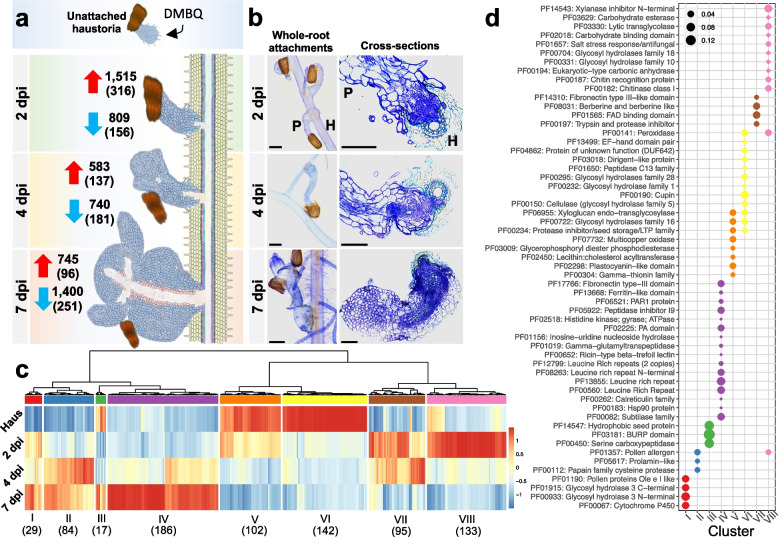
Transcriptome profiling of the *S. hermonthica* secretome during parasitism. **a** Schematic of sample collection for *S. hermonthica* grown on the susceptible rice cultivar, NERICA 7. At each stage the number of upregulated (red arrow) or downregulated (blue arrow) genes are shown relative to the preceding stage (i.e. 2 dpi vs haustoria; 4 dpi vs 2 dpi; 7 dpi vs 4 dpi). Numbers of genes encoding predicted secreted proteins are in parenthesis. dpi = days post infection. **b** Representative microscopy images of whole-root *S. hermonthica* attachments (left) and cross sections of parasite-host attachments on NERICA 7 (right). Scale bars = 100 µM. **c** Hierarchical clustering of FPKM values for the 788 *S. hermonthica* putative secreted DEGs identified from parasites infecting NERICA 7. Numbers in parenthesis represent the numbers of DEGs in each cluster. Scale represents the standardised expression values as a Z-score. d, Enriched Pfam domains for the DEG clusters I-VIII. Point size is proportional to the relative frequency of the domain in the cluster calculated as a proportion of all the domains in that cluster

To achieve our second aim, to identify candidate VF-encoding genes in *S. hermonthica*, we undertook an in-depth analysis of the transcriptional changes for the *S. hermonthica* secretome. Genes involved in parasitism typically show upregulated gene expression during host infection and establishment [9, 22, 28, 33, 34]. Thus, we conducted global transcriptomics profiling of gene expression during host penetration and haustorial establishment stages of the parasite’s life cycle during infection of a susceptible host cultivar, NERICA 7 (Fig. 4). *S. hermonthica* parasites had begun to penetrate the host xylem vessels by 2 days post infection (dpi), and by 7 dpi had a well-developed vascular system with the beginnings of leaf development (Fig. 4a and b). The greatest upregulation of gene expression occurred at 2 dpi relative to unattached haustorial samples, whilst the greatest downregulation occurred at 7 dpi relative to 4 dpi, indicating a wave-like pattern of gene expression coordinated with the penetration process (Fig. 4a).

Of the differentially expressed genes (DEGs) identified, 788 were predicted to encode secreted proteins (i.e. found within the secretome) (Additional File 9). Of these, 80 genes were not annotated with any known Pfam domain, whilst many other genes were annotated with the same Pfam domain(s), suggesting multiple members of gene families are differentially expressed during parasitism. For example, there were 30 DEGs annotated as peroxidases, and 42 DEGs that contained a xylanase inhibitor domain (Additional File 9). We conducted hierarchical clustering of the DEGs and defined eight gene clusters (I – VIII) with distinct expression patterns (Fig. 4c). The gene clusters showed differences in functional enrichment according to their Pfam domains (Fig. 4d, Additional File 10). For example, cluster VIII, which represented genes upregulated during early host penetration (2 dpi) followed by downregulation at 4 dpi, was enriched for glycosyl hydrolase (GH) domains 10 and 18, and a carbohydrate binding domain, as well as two domains commonly found in expansin-like proteins (a lytic transglycolase and pollen allergen domain). Whilst clusters V and VI, which were most highly expressed within in vitro haustoria and were then downregulated in parasites penetrating host roots, were enriched for a different set of GH domains (e.g. GH 1 and 28 in Fig. 4d**,** Additional File 10). Many of these annotations are typical of genes encoding proteins involved in the modification of cell walls and could potentially be involved in modulation of parasite and / or host cell walls to facilitate the penetration process.

Beyond the breakdown / modification of host physical barriers like cell walls, parasitic plants must maintain a compatible interaction through the evasion or suppression of host defences. Accordingly, other DEGs identified through this analysis that are less likely to be involved in the modulation of cell walls may still play an important role in parasitism through functions such as host immune suppression. One potential example of this is provided by a putative secreted gene belonging to the calreticulin family (SHERM_16403), which was strongly upregulated at 7 dpi (cluster IV) (Fig. 4c and d, Additional File 9) and shares similarity with secreted calreticulin effectors delivered by plant parasitic nematodes, which act to suppress host immunity [35].

**Fig. 5:**
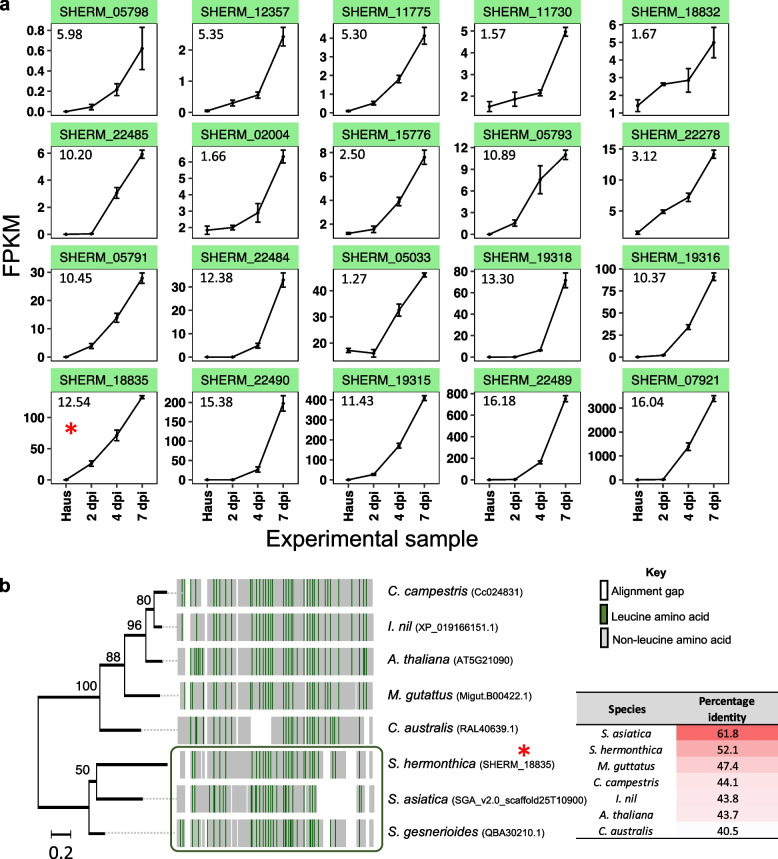
Analysis of leucine rich repeat containing proteins found in cluster IV. **a** Expression profiles for 20 *Striga hermonthica* genes encoding proteins with at least one leucine rich repeat (LRR) domain, which were significantly upregulated at 7 dpi when grown on NERICA 7 (present in cluster IV in Fig. 1c). Plots are ordered according to expression intensity (measured by Fragments Per Kilobase Million (FPKM)) at 7 dpi, from lowest (top left) to highest (bottom right). The red asterisk indicates one of the most strongly upregulated genes (SHERM_18835, log_2_(fold change) = 12.54) that was the closest match to SHR4z, a virulence factor from the closely related species, *S. gesnerioides*. Numbers in the top left of each plot denote the log_2_(fold change) at day 7 compared with the pre-attachment haustoria stage (as determined by DESeq2). **b** Maximum likelihood tree and multiple sequence alignment of the closest match found after searching the SHR4z protein sequence against the proteome of each plant species included in this analysis. Numbers at branch nodes represent bootstraps for 50 replications. Leucine amino acids are highlighted green in the alignment and show a more similar pattern of distribution for the three *Striga* species. The table insert shows amino acid percentage identity for each protein match against the SHR4z

Among the DEGs identified in the secretome, leucine-rich repeat (LRR) domain-containing genes were strongly enriched in cluster IV, indicating that these genes were upregulated later during parasitism (between 4–7 dpi) (Fig. 4c and d). In total, 20 genes from cluster IV (> 10% of the gene cluster) encoded putative secreted proteins with at least one LRR domain (Additional File 9). The SHR4z effector recently identified from the closely related parasitic plant, *S. gesnerioides*, was also annotated as an LRR-containing protein [19]. Given this recent finding, and the abundance of LRR domains identified in cluster IV of our transcriptome, we conducted a BLAST search of SHR4z against the entire *S. hermonthica* proteome. The best matching *S. hermonthica* protein (gene ID: SHERM_18835; BLASTp e-value: 5.52e^−60^; sequence identity: 52.1%) was in fact also present within the set of 20 LRR-encoding genes in cluster IV (Fig. 5a**)**. Moreover, this gene was among the five most strongly upregulated genes, although there were also four other LRR-encoding genes in this cluster with higher fold increases in expression (Fig. 5a). The *S. hermonthica* sequence, SHERM_18835, belonged to orthogroup OG0017883 (orthogroup analysis described above), which contained just two sequences among the seven plant species analysed in this study: one from *S. hermonthica* and one from *S. asiatica*. Thus, these two sequences, and SHR4z from *S. gesnerioides,* are distinct from other sequences in the closely related species, *M. guttatus*. This was confirmed by identifying and aligning the closest SHR4z match from each of the plant species analysed (Fig. 5b). Although all sequences were aligned with identities > 40%, the *Striga* proteins had higher sequence identities and appeared most like each other in terms of their pattern of LRRs (Fig. 5b). In summary, we identified 788 putatively secreted DEGs from the *S. hermonthica* genome that provide evidence for functionally distinct sets of genes expressed during different stages of the parasitism process. This has identified gene families potentially involved in overcoming either physical plant barriers (e.g. proteins with cell wall modification) or in suppressing host immune responses (e.g. calreticulin-like and LRR-containing proteins).

### A large portion of the differentially expressed gene set identified during parasitism was also found as part of the *Striga*-specific secretome

Of the proteins within *Striga*-specific OGs, exactly half (172) were derived from *S. hermonthica* (the others from *S. asiatica*)*,* and of these 115 (67%) were differentially expressed in our transcriptomics dataset (Additional File 11), which is a significantly greater number than expected by chance (Chi-squared = 26.23, df = 1, *p*-value = 3.02e^−07^). This gene set included eight LRR-like genes, including SHERM_18835, the closest match with the effector, SHR4z (Fig. 5a and b). It also includes the LRR-like gene, SHERM_07921, which showed no expression in haustorial samples (average FPKM = 0.05) but by 7 dpi the expression level was far higher than any other gene in this set (average FPKM = 3,399; Fig. 5a, Additional File 11).

**Fig. 6:**
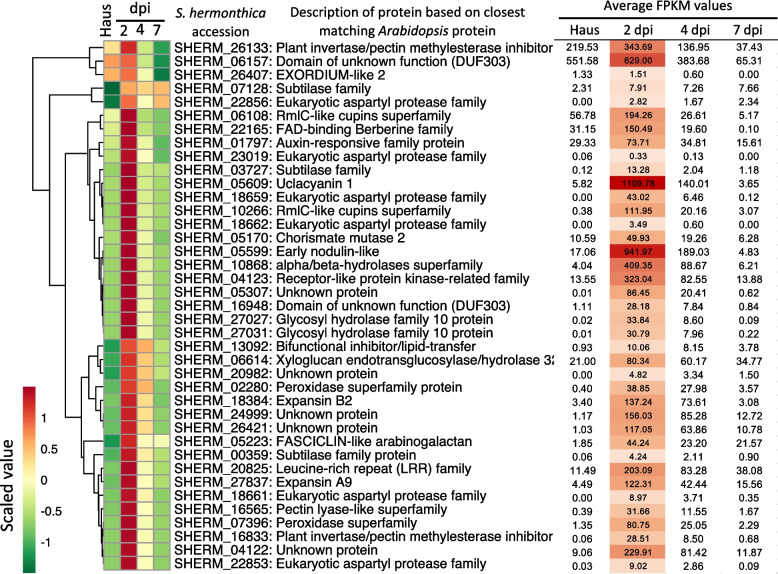
Differentially expressed genes that peak in expression at 2 dpi and which are found in *Striga*-specific OGs. A clustered heatmap of average Fragment Per Kilobase Million (FPKM) values for the 39 genes encoding putative secreted proteins that were differentially expressed with a peak in expression at 2 dpi, and which were found in *Striga*-specific OGs. To the right of the heatmap the average FPKM values are given. The FPKM values for 2 dpi are coloured to highlight the magnitude of expression at this time point, with dark red representing the most highly expressed genes in the set, and pale orange the least

We further refined this set of DEGs to focus on those that peaked in expression at 2 dpi, representing a wave-like pattern of gene expression, which is expected for genes involved in the very early penetration process of the host plant. Of the 115 *S. hermonthica* DEGs found in *Striga*-specific OGs, 39 showed this wave-like pattern of expression (Fig. 6). The genes with the highest expression values at 2 dpi included a gene with homology to *Arabidopsis* proteins annotated as ‘uclacyanin 1’, ‘early nodulin-like protein’, Domain of unknown function (DuF3030) and an ‘alpha/beta hydrolase superfamily’ (Fig. 6). There were also DEGs in this set with the annotation ‘expansin B2’ and ‘chorismate mutase 2’, both of which have been suggested to function as VFs in parasitic plants of *the* Orobanchaceae family [22, 36]. Thus, this set of 39 genes from *Striga*-specific OGs that are upregulated during early penetration and encode putatively secreted proteins represent excellent candidate VFs that are involved in the infection of the susceptible rice host cultivar, NERICA 7.

**Fig. 7:**
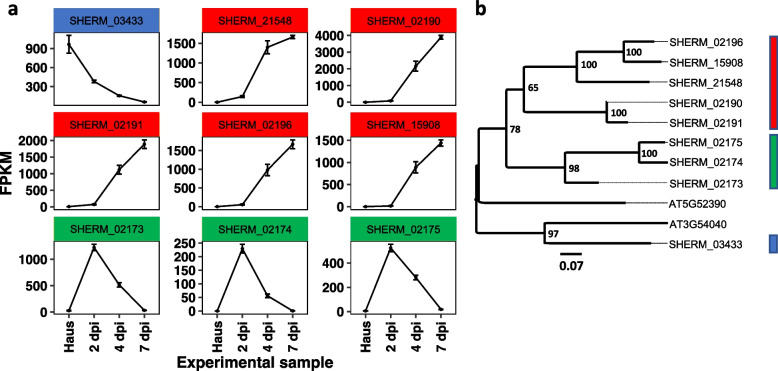
Expression profiles for nine PAR1-like identified in the *Striga hermonthica* secretome. **a** Average Fragment Per Kilobase Million (FPMK) values for each PAR1-like gene at the four experimental collection points. **b** Maximum likelihood phylogeny of the nine PAR1-like proteins from the *S. hermonthica* secretome with two homologs from *Arabidopsis thaliana* (identified by BLASTp). The colored bars correspond to the colors in Fig. 7a, and indicate three PAR1-like gene clusters that display distinct expression profiles during parasitism. Scale represents the proportion of amino acid substitutions per site. Numbers at branch nodes represent likelihood values for 100 bootstrap replicates

Although not specific to *Striga* OGs, we also found that PAR1-like domains were strongly associated with *Striga* secretomes (Fig. 7b). Inspection of the expression profiles for the genes annotated with this PAR1 domain were among the most differentially expressed in *S. hermonthica* during infection of NERICA 7 (Fig. 7a). Of the nine genes with this domain found in the *S. hermonthica* secretome, eight matched the *Arabidopsis* protein AT5G52390, suggesting gene duplication occurred in the lineage leading to *S. hermonthica* since its divergence with *Arabidopsis*. All eight genes were differentially regulated during infection of NERICA 7; five showed increasing expression from 2–7 dpi (cluster IV), whilst the remaining three genes peaked in expression at 2 dpi, then declined by 4–7 dpi (cluster VIII) (Fig. 7a). In contrast with the two *Striga* species, the *Cuscuta* species had a dearth of PAR1 domains present in their secretomes, with zero or one PAR1 domain being identified for *C. australis* and *C. campestris,* respectively, whilst *I. nil* had 16 PAR1 domains in its secretome (Additional File 7). Thus, it seems that the PAR1-like gene family in *Striga* might provide a particularly interesting avenue for research with respect to potential VF function.

## Discussion

### Identification of parasite sets distinct from non-parasitic relatives revealed the types of proteins more strongly linked with *Striga* or *Cuscuta* genera

The availability of genome assemblies for two members of the *Striga* and *Cuscuta* genera allowed us to conduct comparative analyses between these parasitic plant species to identify shared and distinct genes / gene families within the proteome and the secretome. Given that parasitism in *Striga* and *Cuscuta* arose independently and that these two genera parasitise the root and shoot of their hosts, respectively [1], it was surprising to find parasitic plant protein sequences from *Striga* and *Cuscuta* species that clustered together without representative sequences from their non-parasitic relatives clustering in the same OG. Specifically, we found 85 OGs containing proteins from at least three of the parasitic plant species and none from either of the closely related non-parasitic plants. These OGs represented clusters of similar protein sequences that exist despite the independent evolution and distinct modes of parasitism for the *Striga* and *Cuscuta* genera, indicating possible convergent evolution of parasitic plant proteins, or acquisition of such genes through horizontal gene transfer (HGT). One OG from this set had representative protein sequences from all four parasitic plant species and consisted almost entirely of putative secreted proteins (seven secreted proteins of a total of eight in the OG), and was annotated with the domain, ‘strictosidine synthase’ (Table 1; Fig. S2). Strictosidine synthase-like (SSL) enzymes are involved biosynthesis of terpenoid indole alkaloids, which are secondary metabolites that play a role in numerous aspects of plant development and stress responses [37]. Intriguingly, strictosidine synthase-like (SSL) genes were identified in *Orobanche aegyptiaca* and *Cuscuta australis* genomes and were proposed to have been acquired through HGT from a Brassicaceae host plant [38]. This conclusion was based on the higher similarity of the nucleotide sequences with *Arabidopsis thaliana* (a species closely related with the *Brassica* host plants of both parasitic plants) than sequences from the close relatives of the parasitic plants [38]. In agreement with this study, our OG analysis found SSL proteins exist among *C. australis*, *C. campestris*, *S. asiatica* and *S. hermonthica* that share higher amino acid similarities than their non-parasitic relatives. Moreover, our data suggest these proteins may have the potential to be secreted into plant host tissues, where they may interfere with host secondary metabolism. Since these enzymes are involved in monoterpenoid indole alkaloid metabolism, it is possible that secreted SSL enzymes change the metabolic flux through the host’s strictosidine pathway, thus interfering with the production of host defensive secondary metabolites. In line with this, *Cuscuta gronovii,* grown on *Arabidopsis thaliana* mutants unable to produce indole glucosinolates, showed enhanced growth suggesting these compounds can inhibit parasitic plant growth [39]. Overall, these findings suggest a possible role for SSL genes in core parasitic processes of shoot and root parasitic plants.

More broadly, our analysis of Pfam domain abundances allowed us to identify domains that were most strongly associated with either the *Striga* or *Cuscuta* secretomes, relative to the autotrophic plants in this analysis. The three domains most highly associated with *Striga* species were ‘serine carboxypeptidases’, ‘PAR1’ and ‘pollen proteins Ole’ domains (Fig. 3a). A previous comparative transcriptomics analysis of three root parasitic plants in the Orobanchaceae family (*Triphysaria versicolor*, *S. hermonthica* and *Phelipanche aegyptiaca*) identified the ‘serine carboxypeptidase’ domain as highly enriched in haustoria-expressed transcripts [33]. Moreover, signatures of positive selection were found in an orthogroup containing proteins annotated as PAR1-like proteins [33]. Yang et al. (2015) [33] also concluded that genes involved in pollen tube growth and development were likely co-opted during the transition to parasitism in the Orobanchaceae family, which may explain the identification of the ‘pollen proteins Ole’ domain (PF01190) in our analysis (Fig. 3a). That this domain was not also identified to be more abundant in the *Cuscuta* secretomes could indicate that *Cuscuta* species did not co-opt genes involved in pollen tube development during their transition to parasitism, as suggested for parasitic members of the Orobanchaceae [33]. Furthermore, specifically in the *Striga* parasite set, we also noted an enrichment in proteins with the subtilase domain annotation (PF00082) (Fig. 3b), including three genes that were specifically upregulated at 2 dpi (Fig. 6). In line with this, *Striga spp*. upregulate numerous subtilase genes upon infection [40]. Furthermore, a previous study showed upregulation of a collection of genes encoding secreted subtilase proteins specifically in intrusive cells in *Phtheirospermum japonicum*, which like *Striga spp*., is a member of the Orobanchaceae family [41]. In an elegant experiment, the authors demonstrated that inhibition of these *P. japonicum* subtilases reduced the rate of successful differentiation of intrusive cells, and they thus concluded these proteins play a role in haustorium development. Since intrusive cells are in direct contact with host tissues, and the subtilase proteins were predicted to be secreted, it is feasible that these proteins interact with components of parasite or host biology to facilitate successful differentiation of parasite haustoria.

The function of PAR1-like proteins in *S. hermonthica* is difficult to predict based on the available data for the homologs in *Arabidopsis* (AT5G52390 and AT3G54040). According to the *Arabidopsis* developmental transcriptome map [42](see also TAIR entry for AT5G52390), the gene AT5G52390 is most highly expressed in the senescent leaf and the sepals of the developing plant. However, we found that *S. hermonthica* has eight orthologs to AT5G52390 (Fig. 7b), with two distinct gene expression patterns during parasitism on NERICA 7 during infection. This suggests gene duplication and possible evolution of novel functions of some paralogs may play a role in parasitism, although the exact functions of different PAR1-like paralogs in *Striga spp*. remain to be elucidated.

In *Cuscuta* species, there was a strong association with GMC oxidoreductase domains within the secretome. These domains are found in proteins belonging to a superfamily of oxidoreductase enzymes found in fungi, bacteria, insects and plants [43], and have become the focus of research due to their potential role in industrial lignocellulose degradation [43, 44]. A subgroup of this family is classified as glucose oxidases, an example of which was found in the saliva of the herbivore, *Helicoverpa zea*, to dampen herbivore-induced host defences [45]. Other GMC oxidoreductases have been identified as a major component of the aphid salivary sheath and are possibly involved in quenching host reactive oxygen species or detoxifying phytochemicals [46]. Perhaps this protein domain is important for phloem-feeding parasites (e.g. aphids and *Cuscuta* spp.).

### The *S. hermonthica* transcriptome identified families of differentially regulated genes encoding secreted proteins, some of which likely represent VFs

In this study we profiled changes in the *S. hermonthica* transcriptome during the parasitism of a susceptible host rice cultivar, NERICA 7. It is abundantly clear from this and other studies that plant cell wall modification represents an important aspect of forming a successful connection with a host plant [11, 20–22, 33, 47]. Cell wall modifying proteins often possess carbohydrate active enzyme (CAZyme) activity and are attributed a glycosyl hydrolase (GH) family number depending on the precise mode of enzyme activity [48]. In our study, we found different GH-containing genes had distinct expression profiles. For example, genes encoding secreted proteins with GH10 or GH18 domains were expressed at very low levels in haustoria, were highly expressed at 2 dpi and were downregulated thereafter (cluster VIII in Fig. 4c). In another example, a cluster of genes that included those with GH3 domains displayed low levels of expression in haustoria and were then upregulated at all attachment stages, but particularly at 7 dpi (cluster I in Fig. 4c). Qiu et al. (2022) [20] recently conducted an experiment to search for signatures of allelic differentiation between parasite populations collected from NERICA 7 and the very few successful parasite attachments collected from a more resistant rice line, NERICA 17. An *S. hermonthica* gene containing a GH3 domain (SHERM_20042) had a strong signature of allelic differentiation within the promoter region, suggesting differences in expression of this gene can contribute to overcoming the resistance found in NERICA 17 [20]. In another study, comparative transcriptomics analysis of parasitic plants from the Orobanchaceae family was used to identify genes likely involved in parasitism, including several GH-containing genes [33].

Interestingly, there were other genes encoding proteins with GH domains that were highly expressed in in vitro-grown haustoria, but which were downregulated during interaction with the host (see cluster VI in Fig. 4c). An example would be genes encoding proteins containing the GH5 domain, found in some cellulases, which may be involved in coordinating cell growth / shape change during haustorial development. One potential consequence of cell wall modification is the release of small molecules, such as oligosaccharides, and such perturbations can release Damage Associated Molecular Patterns (DAMPs) that provide a mechanism to alert the plant’s immune system to parasitic invasion [49]. Thus, our data suggest that, in addition to the upregulation of a set of cell wall modifying genes that act as VFs to aid host penetration, the controlled downregulation of others may also be necessary to the infection process, possibly to avoid unwanted release of DAMPs that could trigger the host immune response. Indeed, it has been noted that biotrophic fungal plant pathogens and mutualistic fungi possess the fewest CAZymes, whilst necrotrophs have the most [48, 50], suggesting that fungi interacting with living host tissues may restrict their cell wall modifications, thus reducing the chances of releasing DAMPs. Our finding that parasitic plants appear to have reduced secretome sizes could also be explained by the need to carefully control the release of potential DAMPs to prevent triggering a host immune response. Yet in parasitic plant–host systems, the parasitic plant must retain genes encoding cell wall modifying enzymes required for its own growth and development, as well as maintaining sets of genes geared towards host cell wall manipulation. Thus, highly controlled regulation of sets of cell wall modifying proteins is likely to be important for parasitic plants, and mis-regulation of a particular gene at a given developmental stage could trigger the recognition by the host immune system. In line with this, 33 out of 38 putative secreted VFs identified by Qiu et al. (2022) [20] contained signatures of allelic differentiation within the promoter region of the gene, highlighting the likely importance of gene regulation in forming successful parasite attachments. Highly controlled gene regulation is perhaps more important for parasitic plants with a broad host range, like *S. hermonthica*, which may encounter many different host species / varieties, each differing slightly in their cell wall composition. It would thus be very informative to generate comparative transcriptomics datasets during interactions with different host species and varieties, and for parasitic plants with broad versus narrow host ranges.

As well as evading a host immune response through controlled regulation of parasite gene expression, it is expected that parasitic plants like *S. hermonthica* will deploy other VFs that suppress the plant’s immune system. In contrast to cell wall modifying proteins, which are likely to function mainly during penetration and thus follow a wave-like pattern of expression, VFs that suppress the immune response may follow a different expression pattern that is sustained even at later time points, as these genes would potentially need to be expressed for the entire duration of a compatible interaction with the host plant. In the present study, we identified DEGs from *S. hermonthica* that increased from 2/4 dpi to 7 dpi, and may therefore capture such late-acting, immune-suppressive VFs. The putatively secreted LRR-containing proteins identified from *S. hermonthica* are interesting in this regard for three reasons. Firstly, we found a strong enrichment of LRR-encoding genes that were progressively upregulated from 2–7 dpi. Secondly, one of the LRR-genes in this cluster showed close homology of one protein with the recently described immune-suppressing effector from *S. gesnerioides* [19]. Finally, many of these LRR genes only displayed expression when in contact with the host, displaying very low or no detectable expression within in vitro haustoria (Fig. 5a), suggesting that the induction of their expression may require a signal from the host plant. LRR-genes encode a large and diverse class of proteins involved in the recognition of molecular patterns (proteins or small molecules) and play a particularly prominent role in plant immune responses [19, 51, 52]. Such proteins represent a source of molecular diversity that could provide the basis for evolution of secreted VFs, which are delivered into host plants to interact with components of the host’s immune system, as was found by Su et al. (2019) [19] for *S. gesnerioides* infecting cowpea.

Within our transcriptome we found gene families with similar expression profiles; the genes encoding LRR-containing and PAR1-like proteins providing good examples. Why would a parasitic plant upregulate multiple gene paralogs in a similar manner? One possibility is that the different paralogs have similar temporal expression profiles yet are spatially distinct in the developing parasite. Alternatively, this apparent redundancy could be explained if different paralogs are involved in the interaction with different hosts. If this is true, our evidence of multiple LRR-containing or PAR1-like genes expressed in a similar manner suggests that at least some gene families in *S. hermonthica* are not transcriptionally responsive to the host cultivar but rather are expressed in a fixed manner, regardless of host. However, other data show this is not true for all parasite genes / gene families, as examples of host-specific transcriptional regulation already exist [22]. A similar explanation for genetic redundancy has been given for the array of KAI2 / HTL α/β-hydrolase receptor proteins encoded within the *S. asiatica* and *S. hermonthica* genomes, which have been reasoned to exist in parallel to perceive different strigolactones derived from host plants [40, 53, 54]. The existence of such genetic redundancy as an adaptation to enable the parasitism of multiple hosts raises the possibility of using redundancy as an additional criterion with which to identify genes potentially encoding VFs proteins involved in the interaction with different host plants.

### The cross-section of *S. hermonthica *DEGs that were found in *Striga*-specific OGs revealed excellent candidate VFs

Our OG analysis identified gene families specific to *Striga* (*Striga*-specific OGs). Further, we found a significant proportion (67%) of the *S. hermonthica* genes encoding putative secreted proteins from these *Striga*-specific OGs were also differentially expressed in our transcriptome during parasitism of NERICA 7. By focusing our analysis on those genes displaying a wave-like pattern of expression, as expected for VFs involved in the early penetration stages of parasitism, we identified a small set of genes (39) that represent candidate VF for future characterisation. It is noteworthy that our 39 candidate VFs identified here shared no overlap with a set of 38 predicted VFs identified from the same accession (Kibos) of *S. hermonthica* using a population-genetics approach [20]. However, this is perhaps not unexpected. In our study, we infected a highly susceptible rice cultivar (NERICA 7) with *S. hermonthica* and looked for genes specifically upregulated at 2 dpi, whilst Qiu et al. (2022) [20] identified candidate VF from a sub-population of the Kibos accession of *S. hermonthica* that were able to overcome the strong resistance found in the rice cultivar, NERICA 17. Specifically, to be identified by Qiu et al*.* (2022) [20], there had to be polymorphism with alleles responding differently to the two host cultivars. This necessarily misses VFs that are needed for penetration of all hosts and are not polymorphic. Thus, the distinct sets of putative secreted VFs found by these two approaches highlight the differences obtained when focussing only on a susceptible host (this study) versus a comparative analysis between parasites infecting susceptible and resistant host cultivars [20].

Among our 39 candidates, the two genes with the highest expression values at 2 dpi were annotated as ‘uclacyanin 1’ and ‘early nodulin-like’ (Fig. 6). Notably, both these genes fall into different sub-families of phytocyanins (blue copper proteins), which have a range of assigned roles in plant growth and development [55]. Uclacyanin proteins, for example, are involved in the lignification of the Casparian strip [56]. Nodulin-like proteins are not only important for establishing mutualistic symbioses but are also implicated in susceptibility to pathogens [57]. Furthermore, at least two of the genes we identify here have functional annotations like those previously implicated in plant-plant parasite interactions: the ‘expansin B2’ and ‘chorismate mutase 2’ gene (Fig. 6). A β-expansin is specifically and highly up-regulated in haustorial tissue when infecting *Zea mays* but not *Medicago truncatula* [22]. In the latter case, secreted chorismate mutase VFs have been identified from nematode and fungal plant pathogens [36, 58]. These chorismate mutase proteins are delivered into host cells and interfere with the salicylic acid biochemical pathway leading to reduced salicylic acid production, which in turn increases virulence of the pathogen. In our study, a putative secreted chorismate mutase from *S. hermonthica* (SHERM_05170) showed upregulation at 2 dpi followed by downregulation at later time points (Fig. 6). Similarly, the expression of the Cmu1 VF from *Ustilago maydis* is specifically upregulated during biotrophic host invasion [36].

### Conclusions and future directions

In this study, we capitalised on the increasing genomic resources for parasitic plants to achieve two key aims. First, we used comparative orthogroup and protein domain analyses to compare *Striga* and *Cuscuta* secretomes, which led to the identification of commonalities (e.g. strictosidine synthase like enzymes) and differences (e.g. PAR1-like proteins or GMC oxidoreductase-like proteins) between the two genera of parasitic plants. We then conducted a detailed transcriptional profiling of genes encoding putatively secreted proteins for the most agriculturally damaging parasitic weed, *S. hermonthica*, which revealed clusters of DEGs during the initial parasitism stages of a susceptible host plant.

The intersection of these analyses provided a means to select a small set of potential VFs, which represent excellent candidates for future functional validation studies. Going forward, the challenge will be two-fold. Firstly, to verify and determine the molecular mechanism of the most promising candidate VFs that have now been predicted. This is challenging in parasitic plants without a stable transformation protocol but in some cases heterologous expression systems in *Arabidopsis* (or other genetically tractable plants) might provide one avenue for functional validation studies. However, for this to work it will be crucial to develop the correct genetic background. For example, the *Arabidopsis htl-3* loss-of-function line allowed the functional investigation of *Striga HTL* receptors (*ShHTL1-11*) [54]. The second challenge will be to use this information to aid the development of sustainable control strategies and to delay the evolution of virulence in the parasite. To address these challenges, it will be beneficial to determine whether any VFs are essential to parasitic plant survival and if so to target these VFs.

## Methods

### Genome assemblies and annotations

The source and version of each genome assembly and the associated annotation files for species used in this study are given in Additional File 12. To avoid analysing multiple isoforms of the same protein, the primary transcript protein annotation file was used, if available from source. If not available, the longest protein isoform for each protein-coding gene was extracted using a custom Python script. Only proteins larger than 30 amino acids were considered.

### Functional annotation of protein sequences

For each sequence in each proteome, the protein length (number of amino acids) and amino acid composition (percentage content of each amino acid) were calculated (custom Python script). Pfam domain annotations were obtained using InterProScan (version: 5.32–71.0) [59, 60]. The proteome of *Arabidopsis thaliana* is well annotated functionally, and therefore a comparison against the *A. thaliana* proteome was made to obtain more information on the putative protein function. The ‘blastp’ tool was used to query each protein from each species against a database of *A. thaliana* protein sequences, built using the ‘makeblastdb’ tool from the NCBI BLAST package (version: ncbi-2.3.0 +). A BLASTp hit was significant if the e-value < ${10}^{-5}$ with a query coverage of at least 50%. The BLAST analysis of the SHR4z sequence from *Striga gesnerioides* (GenBank accession: MG870386.1) against the proteomes of the seven plant species was conducted in the same way.

### Prediction and analysis of the secretomes

Proteins can be secreted via non-classical or classical pathways, with the latter being directed through the endoplasmic reticulum and Golgi apparatus via an N-terminal signal peptide [61, 62]. The software, SignalP5.0, has recently been benched-marked against 18 other algorithms and performed best for detection of secretion signals from eukaryotic sequences when assessed for the accuracy of true positive predictions of the signal peptide and the prediction of the signal peptide cleavage site within the amino acid sequence [62]. Thus, SignalP5.0 was downloaded and run locally to scan each peptide for the presence of a signal peptide within the first 70 amino acids of each protein. For those proteins with a predicted signal peptide, the mature protein (peptide after removal of signal peptide) was scanned for a transmembrane domain using TMHMM2.0, which could indicate the protein may reside in the plasma membrane [63]. The final secretome predictions consisted of proteins with a signal peptide and which lacked a predicted transmembrane domain in the remaining portion of the peptide (Fig. S3).

To identify significantly enriched Pfam domains in each secretome, the proteome was divided into two categories: the secretome and non-secretome. The numbers of each domain in both categories were counted and a Chi-squared test was applied to determine if the corrected *p*-value was < 0.01 (according to the Benjamini–Hochberg procedure). Enriched Pfam domains found in each secretome were further analysed using a principal component analysis (PCA). First, the abundance of each domain in the secretome was calculated as a percentage of the total number of Pfam domains in the secretome for that species. Pfam domains were included in the clustering analysis if they met the following two criteria: (i) Pfam abundance in the secretome for the parasitic plant species was greater than compared with the non-parasitic relative and (ii) Pfam domain abundance > 0.25% (to remove the long tail of Pfam domains with a very low abundances).

A domain was deemed as ‘highly abundant’ in the secretome of the *Striga* or *Cuscuta* species if the abundance of that domain was > 0.25% of all Pfam domains in both parasitic plant species (i.e. the two *Striga* species or the two *Cuscuta* species) and if this abundance was greater than the corresponding abundance for the non-parasitic relative. A matrix of abundances for these Pfam domains for the four parasitic plants was used as the input into the prcomp function in R [64] (R Core team), with scale and centre set to ‘true’ in order to identify which Pfam domains clustered together in PCA space. The PCA results were visualised as a biplot (plotted using the ggbiplot function in R) for the top four principal components.

### Protein family classification into orthogroups and evolutionary analysis

Orthogroups were defined among the proteomes of the seven plant species (Additional File 12; Fig. S3) using OrthoFinder (version 2.3.7), which was installed locally and run according to the authors’ instructions [65]. A species tree was inferred using STAG (species tree inference from all genes), implemented by OrthoFinder [65]. This analysis allowed a species tree to be obtained where each node was supported by a STAG support value, which represents the proportion of times that a given bipartition in the tree was observed among all the individual species trees estimated from each of the OGs that contained at least one sequence from each species [65]. The tree was rooted using STRIDE, as described by the OrthoFinder manual.

To detect OGs that had expanded or contracted for each branch of the species phylogeny, an ultrametric tree was first constructed. This was achieved using the 287 single-copy OGs, alignments of which were made using MUSCLE (version 3.22) and then concatenated to build a maximum likelihood (ML) phylogenetic tree using MEGA X [66] following the Jones-Taylor-Thornton (JTT) substitution model. The ML tree was then converted to an ultrametric tree using MEGA X and used as input into the software CAFE (version 4.1), along with a matrix of OG protein counts per species, which models the gene birth/death rate, to detect the number of significantly expanded or contracted gene families per branch of the tree, using a *p*-value < 0.001 [67]. The lambda (birth/death rate parameter) was set to 1 for all branches in the phylogeny. Venn diagrams of overlapping orthogroups were created using InteractiVenn (http://www.interactivenn.net/).

The OG counts were divided into groups consisting either entirely of non-secreted proteins (non-secreted OGs), entirely of secreted proteins (secreted OGs) or a mix of non-secreted and secreted proteins (mixed OGs). For the genus and family specific OGs, we sought to test whether there was an enrichment of secreted and mixed OGs in the parasitic branches of the tree (*Striga* and *Cuscuta* genera). To do so, a comparison was made with the proportions of non-secreted, secreted or mixed OGs at the genus and family level using a Chi-squared test (full analysis presented in Additional File 3).

### Selection of *Striga* and *Cuscuta* parasite sets

One difficulty in the identification of putative VFs from parasitic plant secretomes is distinguishing those secreted proteins likely to be involved in parasitism of the host plant from those proteins involved in aspects of the parasitic plant’s own development (although these two groups are not necessarily mutually exclusive). We used the OG analysis described above to try to separate a subset of the secretome more strongly associated with the secretomes of the parasitic plants, which are possibly more likely to play a role in parasitism. Accordingly, subsets of proteins from the *Striga* or *Cuscuta* secretomes were selected that met the following criteria: (i) found in OGs specific to the *Striga* or *Cuscuta* genera (or a single species within these genera), (ii) found in OGs that were expanded within the *Striga* or *Cuscuta* genera (or a single species within these genera), or (iii) were unassigned to any orthogroup for any of the *Striga* or *Cuscuta* species. The resulting sets of *Striga* and *Cuscuta* proteins were deemed the ‘*Striga* Parasite Set’ (SPS) and the ‘*Cuscuta* Parasite Set’ (CPS) (see also step 3 in Fig. S3). These sets of proteins were compared by assessing the differences in abundance of protein domains and presented as a spider plot, where abundance was expressed.

### Growth and collection of *S. hermonthica* attachments

The rice cultivar NERICA 7 displays high levels of susceptibility to the Kibos accession of *S. hermonthica* [68]. This cultivar was infected with pre-germinated (0.1 ppm GR24) *S. hermonthica* seeds (Kibos accession) in rhizotrons, as described in [69]. Plants were grown in a controlled environment growth chamber with an irradiance at plant height of 500 μmol.quanta.m^−2^.s^−1^, a day/night temperature of 28 ^◦^C/24 ^◦^C, a photoperiod of 12 h and a relative humidity of 60%. *S. hermonthica* were collected at 2, 4, and 7 d post infection (dpi) by cutting the rice root either side of the attachment and immediately freezing in liquid N_2_ (Fig. S4). At each time point, four biological replicates were collected, each of which consisted of attachments collected from the roots of two rice plants.

In addition to *S. hermonthica*-*O. sativa* attachments, four biological replicates of unattached *S. hermonthica* haustoria were also collected (Fig. S4). To obtain these, 120 mg *S. hermonthica* seeds were sterilised, conditioned, and germinated as described in [69]. Sixteen hours after the induction of germination by GR24, each Petri dish of seeds was treated with 5 μM 2,6-dimethoxy-1,4-benzoquinone (DMBQ). The seeds were incubated at 30 ^◦^C for a further 16 h. Seedlings were then viewed under the dissecting microscope to confirm haustoria had been induced and were flash frozen in liquid N_2_.

### Microscopy of *S. hermonthica* attachments

To interpret changes in gene expression at different times after infection, individual *S. hermonthica* attachments were harvested from the roots to generate cross-sections for microscopic analysis. Attachments were collected, fixed, mounted, and stained as described by [68]. The sections were imaged at × 10 and × 20 magnification using a DMC4500 DOC camera mounted on a Leica DM5 microscope, using bright field. Whole attachments were also collected and placed into a saturated chloral hydrate solution. After a period of at least 1 w, samples were stained with phloroglucinol, prepared as a 3% solution in EtOH and mixed in a 2:1 ratio with concentrated HCL (two parts 3% phloroglucinol to one-part HCl). Samples were counter-stained with a 0.01% solution of Coomassie brilliant blue for 2 min (ThermoFisher). After staining, whole attachments were placed onto glass slides in 10% glycerol and covered with a glass cover slip. These were imaged using bright field microscopy under a Leica M165 stereomicroscope.

### RNA extraction, DNase treatment and RNA cleaning

Total RNA was extracted using the RNeasy plant mini kit (Qiagen, #74,904) (Fig. S4). RNA was eluted in dH_2_O and the concentration determined using spectrophotometry (Nanodrop-1000). RNA (3 µg) was DNase-treated (dsDNase, Thermoscientific) to remove genomic DNA contamination. The RNA was purified and concentrated using the RNeasy MiniElute Cleanup Kit (Qiagen, #74204). Samples were sent to NovoGene Co. Ltd. (China) for library preparation and sequencing.

### RNA sequencing and mapping reads against the *S. hermonthica *and *O. sativa* reference genomes.

Novogene enriched for mRNA using oligo(dT) beads and randomly fragmented the mRNA using fragmentation buffer prior to cDNA synthesis. The cDNA libraries were sequenced using an Illumina technology (unique identifier of sequencing machine: HWI-ST1276). The raw reads were filtered to remove: (i) reads with adapter contamination, (ii) reads in which uncertain nucleotides constituted > 10% and (iii) reads in which low quality nucleotides (base quality score < 20) constituted > 50%. Raw reads were submitted as fastq files to NCBI under BioProject ID: PRJNA992392. As the samples were a mix of *S. hermonthica* and *O. sativa* tissue, the cleaned reads were mapped to both the *S. hermonthica* genome, Kibos accession [20] and the genome for *O. sativa*, subsp. *japonica*, variety Nipponbare (MSU, version 7), using Tophat2 (version v2.0.12) with default settings, except the ‘mismatch’ option was set to 2. A very small proportion of the total mapped reads (average of 0.16%) mapped to both the *S. hermonthica* and *O. sativa* reference genomes, which was deemed low enough not to affect the downstream analyses.

### Gene expression analysis

Transcript abundance was determined as read counts by HTSeq (version 0.6.1) with default settings, except that the ‘-m’ option was set to ‘union’. Analyses of read counts were carried out using the DESeq2 package (version 1.22.2) [70] in R (version 3.5.1) [64]. A gene was considered expressed if the sum of read counts across all biological samples ≥ 10. Quality assurance checks were first carried out by first transforming the read counts according to the variance stabilisation transformation (vst), which removes the dependence of the variance on the mean [70]. A heatmap of sample-to-sample distances and a principal component analysis (PCA) plot showed high reproducibility in within each treatment (Fig. S5). Differentially expressed genes between treatment groups were identified using the likelihood ratio test within the DESeq2 package [70]. For each gene, the differential gene expression analysis produced a log_2_(fold change) (LFC), a *p*-value of significance and a *p*-value adjusted for multiple testing following the Benjamin-Hochberg false discovery correction procedure. DEGs were deemed significant at adjusted *p* < 0.01 and |LFC|> 1.5. (We used a LFC cut-off of 1.5 as we found large numbers of differentially expressed genes due to the low variation between our biological replicates and the large variation between treatments (see Supp. Fig. S5b)). Differentially expressed genes were subject to hierarchical clustering using FPKM values. A distance matrix was constructed according to the ‘Euclidean’ method and clustering was then performed using the hclust() function in R [64], according to the ‘WardD2’ method. The resulting clustered expression values were then rendered as a heatmap using the pheatmap package (version 1.0.12) in R [64]. For each gene cluster, enriched Pfams were determined as described below and taken to be significant if the adjusted *p*-value ≤ 0.05.

### Supplementary Information

**Supplementary Material 1. **

**Supplementary Material 2. **

**Supplementary Material 3.**

**Supplementary Material 4.**

**Supplementary Material 5.**

**Supplementary Material 6.**

**Supplementary Material 7.**

**Supplementary Material 8.**

**Supplementary Material 9.**

**Supplementary Material 10.**

**Supplementary Material 11.**

**Supplementary Material 12.**

**Supplementary Material 13.**

## Data Availability

All data are present within the manuscript and Additional Files 1, 2, 3, 4, 5, 6, 7, 8, 9, 10, 11, 12. Fastq files arising from transcriptome sequencing have been submitted to the National Center for Biotechnology Information (NCBI) with BioProject ID: PRJNA992392.
