# Supplementary material for: Comparative secretome analysis of Striga and Cuscuta species identifies candidate virulence factors for two evolutionarily independent parasitic plant lineages
Source: BMC Plant Biol. 2024 Apr 6;24:251. doi: 10.1186/s12870-024-04935-7 (PMC10998327; doi:10.1186/s12870-024-04935-7)
Supplement: Supplementary file 13 — Supplementary Material 13. [file 12870_2024_4935_MOESM13_ESM.docx]

**Supplementary Information**

The following supplementary materials are available:

**Supplementary Figures**

| **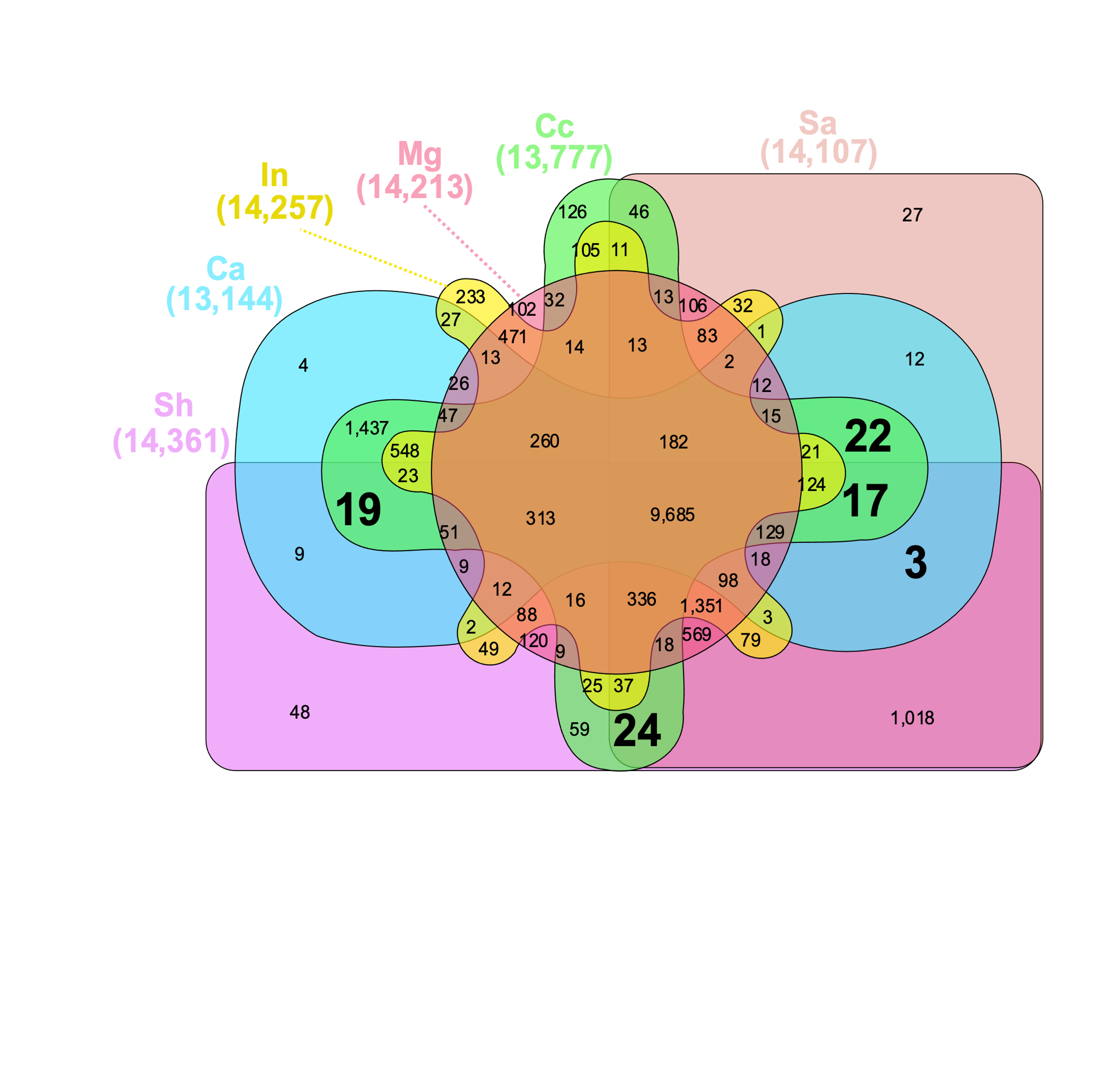** |
| --- |
| **Figure S1**  Overlap in OGs among the four parasitic plants and the two non-parasitic close relatives (excluding *Arabidopsis thaliana*). The bold numbers denote the five sets of OGs that contain proteins sequences from at least three of the four parasitic plants but no protein sequences from the non-parasitic plant relatives. *Sh: Striga hermonthica, Sa*: *Striga asiatica, Mg*: *Mimulus guttatus, Ca*: *Cuscuta australis, Cc*: *Cuscuta campestris,* In: *Ipomoea nil, At*: *Arabidopsis thaliana*. |

| ** |
| --- |
| **Figure S2**  Gene trees from OrthoFinder analysis for seven orthogroups (OGs) that contain protein sequences with the Pfam domain, PF03088—strictosidine synthase. OG0011191 is the only OG consisting of sequences from parasitic plants, whilst all other OGs contain sequences from a mix of parasitic and autotropic plants. Circles to the right of the sequence identifiers indicate whether the protein was predicted to be secreted (red) or not (unfilled). |

|  |
| --- |
| **Figure S3**  The four-step pipeline for the prediction of plant secretomes and identification of sets of ’parasite sets’ of secreted proteins from the secretomes of the parasitic plants. |

| **** |
| --- |
| **Figure S4**  a, Pipeline used for the sample collection, RNA extraction, RNA sequencing and analysis of the reads generated from *Striga hermonthica* attachments*.* Each treatment consisted of 4 biological replicates collected at 2-, 4-, or 7-days post infection (dpi). b, Example of a *S. hermonthica* haustoria induced by DMBQ treatment for 16 hrs. c, Example of a NERICA 7 rice root after 7 dpi with *S. hermonthica*. Insert shows a well-developed attachment. The dashed lines denote approximate cut sites on the host root that were made in order to collect each attachment. Scale bar = 1 mm. Images taken with a DMC4500 camera using the Leica DM6 light microscope. |

| 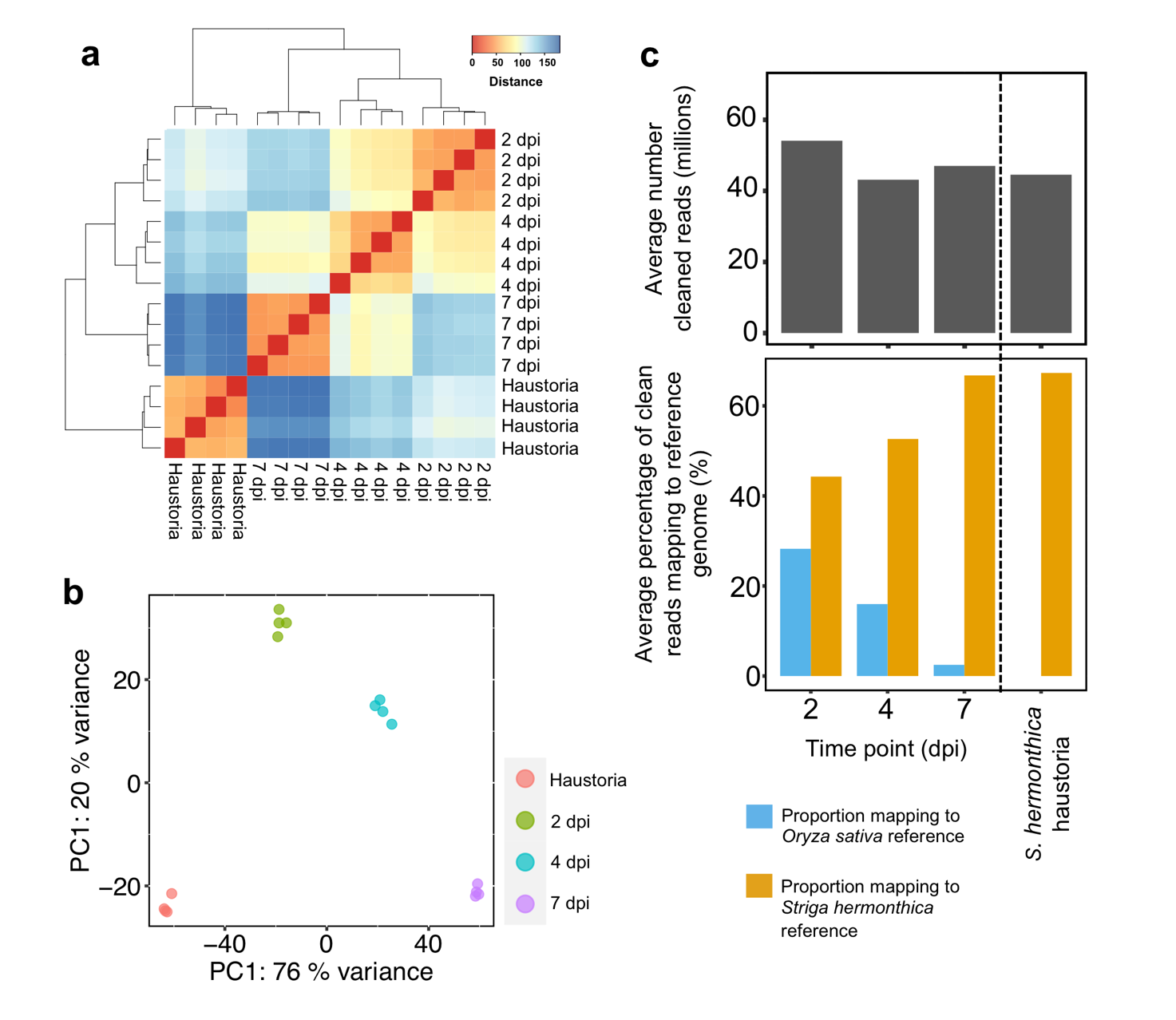 |
| --- |
| **Figure S5**  a, Heatmap of the sample-to-sample distances for each biological sample mapped to the *Striga hermonthica* (Kibos) reference genome. The distance matrix was created using the Euclidean method and plotted with heatmap.2 in R [64]. b, Principal components analysis conducted using the plotPCA function in R showing the relationship between each biological sample for the first two principal components. c, Average number of cleaned reads generated per treatment (upper plot) and the proportion of reads mapped to either the *S. hermonthica* reference genome or the *Oryza sativa* Nipponbare reference (lower plot). dpi = days post infection. |
